# New Mouse Lines That Drive Tetracycline-Controlled Gene Expression in a Small Subset of Spinal Cord Dorsal Horn Neurons

**DOI:** 10.1523/ENEURO.0441-24.2025

**Published:** 2025-04-18

**Authors:** Eric Fyrberg, Heather Learnard, Soojin Lee, Yong-Woo Jun, Fen-Biao Gao

**Affiliations:** ^1^RNA Therapeutics Institute, University of Massachusetts Chan Medical School, Worcester, Massachusetts 01605; ^2^Department of Neurology, University of Massachusetts Chan Medical School, Worcester, Massachusetts 01605

**Keywords:** HB9, interneurons, motor neurons, promoter, spinal cord, tetracycline

## Abstract

Mouse lines with tetracycline-controlled gene expression in specific neuronal populations provide valuable tools for studying their development, function, connectivity, and pathology in vivo. Our initial goal was to generate a mouse model that could express amyotrophic lateral sclerosis-associated genes specifically in spinal cord motor neurons under the control of the *HB9* promoter. However, *HB9-tTA* mice unexpectedly direct target gene expression in a small subset of dorsal horn neurons. These mice represent a new tool for scientists who are interested in studying these spinal cord neurons.

## Significance Statement

We have generated new mouse lines that can manipulate gene expression in a small subset of dorsal horn neurons in the spinal cord. These new tools will be useful for scientists who are interested in studying the development, function, and connectivity of this small subset of spinal neurons in vivo.

## Introduction

Amyotrophic lateral sclerosis (ALS) is an age-dependent neurodegenerative disease characterized by progressive loss of upper and lower motor neurons in the brain and spinal cord, leading to muscle weakness and paralysis (Taylor et al., 2016). One of the key approaches in studying this and similar diseases is the use of animal models that can express toxic proteins in specific cell types to mimic the pathological processes observed in patients (Lutz, 2018). Despite advances in genetic engineering, few mouse models are available that specifically express disease-relevant proteins in motor neurons. The creation of such models will be useful for our understanding how toxic proteins affect motor neurons and contribute to disease progression, as well as for testing potential therapeutic interventions.

The *HB9* promoter, known for its motor neuron specificity (Arber et al., 1999), has been widely used to specifically label motor neurons in cell culture or in vivo (Wichterle et al., 2002; Boulting et al., 2011). In our effort to create a new ALS mouse model in which disease proteins can be expressed only in motor neurons in the spinal cord in a temporally controlled manner, we cloned the *HB9* promoter to drive the expression of a tetracycline-controlled transactivator (tTA), enabling inducible gene expression in the presence of specific tetracycline-responsive elements (Mansuy et al., 1998; Yamamoto et al., 2000; Gascon et al., 2014; Choi et al., 2019).

Unexpectedly, these *HB9-tTA* mice drive target gene expression in a small subset of dorsal horn neurons but not in motor neurons in the spinal cord. Detailed characterization of these mice is described here, and potential reasons for this serendipitous result are discussed. These mice present a new useful tool for scientists who are interested in studying the development, function, and connectivity of this small subset of spinal neurons in vivo.

## Materials and Methods

### Cloning of the *HB9-tTA* plasmid

The sequence encoding tTA was amplified by polymerase chain reaction (PCR) using forward primer GAGGAGGGCGCGCCATGTCTAGATTAGATAAAAGTAAAGTG and reverse primer GAGGAGACCGGTCTACCCACCGTACTCGTC in which we included AscI (5′) and AgeI (3′) restriction sites. The DNA fragment was inserted into the AscI-AgeI sites in the HB9-MCS vector, which was obtained via Dr. Hynek Wichterle from Dr. Thomas Jessell’s lab at Columbia University.

### Generation of Tol2-based *HB9-tTA* transgenic mice

To generate the *HB9-tTA* transgenic mice, we employed a Tol2-based system to induce random chromosomal integration. The *HB9-tTA* cassette was cloned into a Tol2 vector (TG-YK2-001), between Tol2 inverted repeats (IR), and the circular vector was coinjected with Tol2 transposase mRNA into fertilized mouse eggs (C57BL/6J). The transposase facilitated the excision of the *HB9-tTA* DNA fragment flanked by Tol2 IR sites and integrated this fragment randomly into the mouse genome. The injection procedure and handling were performed following the standard protocols provided by Biocytogen.

### Genotyping

Mouse lines from each founder were established by crossing with C57BL/6J mice. We also crossed *HB9-tTA* mice with *tetO-GFP* mice (The Jackson Laboratory, #005104) to detect cells with GFP expression. Approximately 1 mm of the tail tissue was isolated from each mouse following ear tagging for identification. DNA was isolated using the DNeasy Blood and Tissue Kit (Qiagen, 69506), according to the manufacturer's protocol with the exceptions that the digestion step was conducted overnight at 37°C and the final elution was conducted in 100 μl of dH_2_O. Mice were genotyped using the GoTaq Flexi DNA Polymerase reagents (Promega, M8259) with the following primer sequences:

*HB9* Forward: 5′ CCGGTGGAAGTTCATACCAGTGAGT 3′

*HB9* Reverse: 5′ AAGGGCAAAAGTGAGTATGGTGCCT 3′

*GFP* Forward: 5′ GCTCGTTTAGTGAACCGTCAG 3′

*GFP* Reverse: 5′ TCTTCTGCGCCTTAGTCACC 3′

### Brain and spinal cord tissue collection and preparation

Spinal cords and brains were isolated from 2-month-old mice positive for both the *HB9-tTA* transgene and *tetO-GFP* reporter and were perfused with 1× phosphate-buffered saline (PBS) and 4% paraformaldehyde. The spinal cords and brains were fixed by immersion in 4% paraformaldehyde for 48 h at 4°C, then suspended in 30% sucrose, and soaked at 4°C until they ceased floating (∼48 h). The fixed spinal cords and brains were mounted in blocks of a mixture of ∼1/3 OCT (Fisher Scientific, 4585) and ∼2/3 sucrose (30%) and stored at −80°C overnight. The brains and cervical region of the spinal cords were sectioned at a thickness of 20 µm using a Leica CM 1950 Cryostat set to −19°C.

### Immunohistochemistry

Spinal cord and brain sections were washed with 1× PBS and then blocked and permeabilized in 1× PBS containing 3% BSA and 0.2% Triton X-100 (PBST) for 30 min at room temperature. Primary antibodies were applied and allowed to incubate overnight at 4°C. The sections were washed three times with PBST and incubated with secondary antibody at a dilution of 1:2,000 for 2 h at room temperature. Following three washes with PBST, the sections were sealed using ProLong Glass Antifade Mountant with NucBlue Stain (Thermo Fisher Scientific, P36981) and stored in the dark overnight.

The following primary antibodies were used in this study: Elavl2 primary antibody at a dilution of 1:250 (Proteintech, 14008-1-AP) and Anti-Calcitonin Gene Related Peptide (Sigma-Aldrich, C8198) at a dilution of 1:500. The following secondary antibodies were used in this study: donkey anti-rabbit Alexa Fluor 568 secondary antibody (Thermo Fisher Scientific, A10042) and Alexa Fluor 594 goat anti-rabbit IgG (Life Technologies, A11012).

### Quantification of affected cervical spinal cord neurons

Images of the slides containing the cervical spinal cord sections were acquired using a laser-scanning confocal microscope (Carl Zeiss, LSM 800). The resultant images were imported to an ImageJ-based software called Fiji, and the channel containing the endogenous GFP signal was isolated. The isolated channel was converted to a red 8 bit image, and a threshold of 25–255 was set to identify only the endogenous signal while excluding potential background. Using the analysis tools within the Fiji software, the image was measured for individual counts of signal ranging from 100 to 400 pixel units in size, and the resultant output was recorded as the number of GFP-positive neurons per section. This was repeated for five sections per spinal cord, and the results were averaged to yield an average number of GFP expressing neurons per spinal cord.

### Identification of *HB9-tTA* insertion sites in the mouse genome

To identify where the *HB9-tTA* sequence inserted into the mouse genome, fibroblast cells were collected from the ear punch tissue as described (Seluanov et al., 2010; Edelman and Redente, 2018) and cultured in 7 cm tissue culture dishes. The media was changed on Day 3; the cultures were split into two 10 cm tissue culture dishes on Day 6. Media were again changed at Day 9, and cultures were split into four T75 flasks on Day 12. The resulting fibroblasts were harvested on Day 15, suspended in freezing medium composed of 8 ml PBS, 1 ml fetal bovine serum, and 1 ml DMSO (Sigma-Aldrich, D2650), frozen, and then shipped to Cergentis for whole-genome sequencing.

### Body weight measurement

Eight *HB9-tTA* mice (four males and four females) and eight C57BL/6J mice (four males and four females) at the age of 6.8–7.5 months were weighed using a Mettler Toledo PR5002 weighing balance and scale tared to a polypropylene container which served to house the mice during weighing. The resultant weights were analyzed using the GraphPad Prism software via an unpaired *t* test.

### Accelerating rotarod test

Eight *HB9-tTA* mice (four males and four females) and eight C57BL/6J mice (four males and four females) at the age of 5.1–5.8 months had their locomotor function assessed on an accelerating rotarod test. On the day preceding the experiment, all mice were introduced to the apparatus through a training session which involved running on the rotarod for a total of 5 min at 4 rotations/min. Any mice that fell during the training session were placed back onto the apparatus throughout the 5 min session. The mice were given a 10 min rest period between trainings, for a total of three trainings.

On the day of the experiment, the mice were placed on the rotarod rotating at 4 rotations/min, and the latency to fall off the rod was recorded while it accelerated at a rate of 7.2 rotations/min over the course of 5 min, reaching a maximum speed of 40 rotations/min. The mice were given a 10 min rest period between trials, for a total of three trials. The resultant data were imported to the GraphPad Prism software for subsequent analysis via an unpaired *t* test.

### RNA extraction and real-time quantitative PCR

Frontal cortex tissues from mice were collected in 1.5 ml tubes following perfusion with PBS. Total RNA was extracted using TRIzol reagents (Qiagen, 79306) and further purified with the RNeasy Mini Kit (Qiagen, 74106). cDNA was synthesized from 1 µg of RNA using the TaqMan Reverse Transcription Kit (Thermo Fisher Scientific, N8080234). Quantitative real-time PCR was performed using SYBR Select Master Mix (Thermo Fisher Scientific, 4472918) on a QuantStudio 3 System. The primers used were *tTA* (forward, 5′-GACGAGCTCCACTTAGACGG-3′; reverse, 5′-CCCCCAACATGTCCAGATCG-3′), *Myh11* (forward, 5′-GGCTAGCAGCTTGTCAGGAA-3′; reverse, 5′-ATGTTGCCCTGCTCTTCCTC-3′), and *GAPDH* (forward, 5′-AACTTTGGCATTGTGGAAGG-3′; reverse, 5′-ACACATTGGGGGTAGGAACA-3′). Ct values were normalized to GAPDH, and relative mRNA expression levels were calculated using the ΔΔCt method.

## Results

### Characterization of the *HB9* promoter sequence inserted into the mouse genome

**Figure 1.**
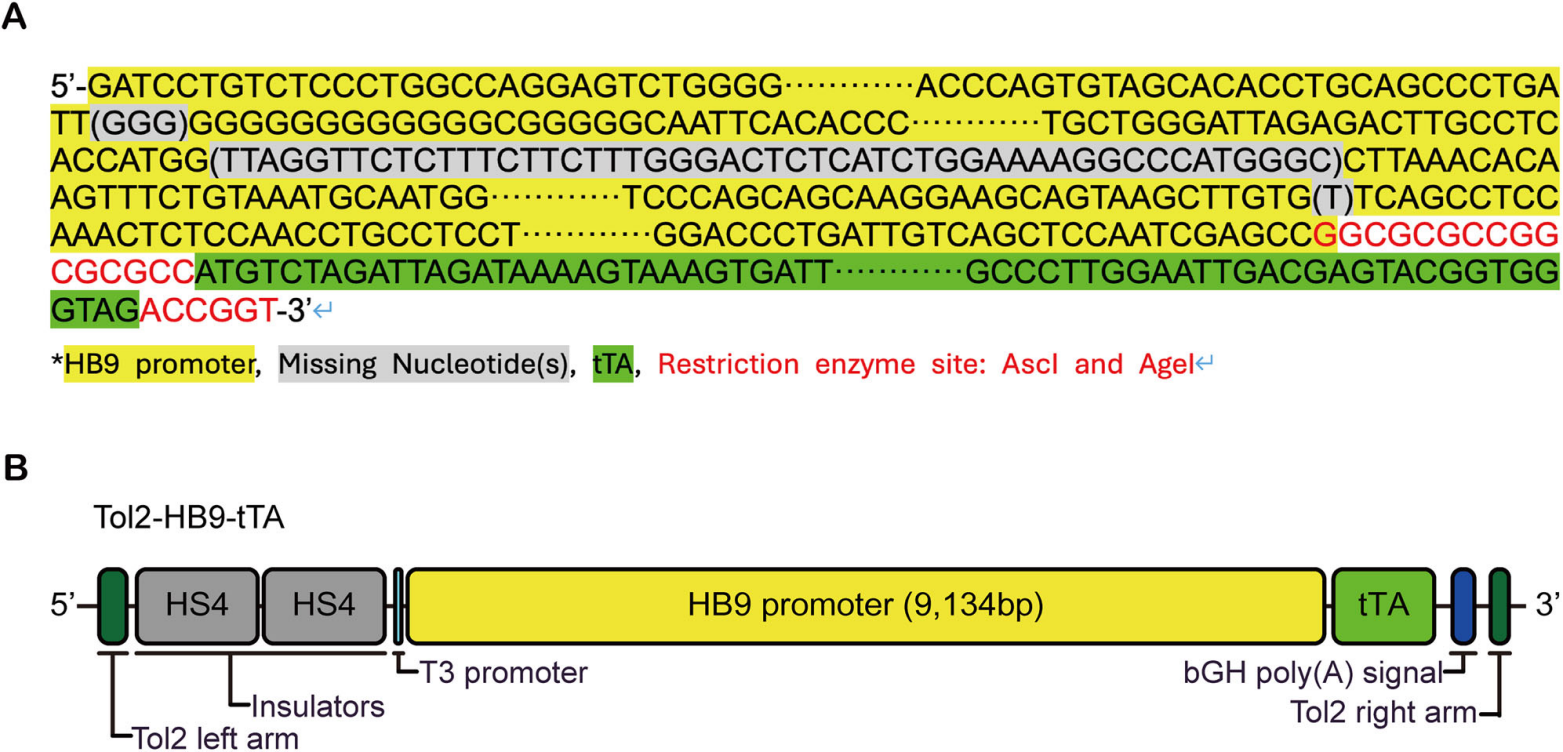
Schematic representation of the *HB9* promoter cassette. ***A***, Nucleotide sequence of the mouse *HB9* promoter and *tTA*. The identity of different sequences is indicated by their specific colors. ***B***, Schematic of the *Tol2-HB9-tTA* plasmid used for generating transgenic mice. To utilize the Tol2 transposon system, Tol2 arms are placed at both ends of the *HB9-tTA* sequence. After the Tol2 left arm, the construct contains HS4 insulators, the mouse *HB9* promoter, *tTA*, and the *bGH* poly(A) signal. Nucleotide sequence of the *Tol2-HB9-tTA* plasmid is shown in Extended Data Figure 1-1.

We obtained the *HB9-MCS* plasmid (Wichterle et al., 2002) and inserted a DNA fragment encoding the tTA protein flanked by AscI and AgeI restriction enzyme sites. DNA sequencing analysis revealed that the *HB9* promoter in the *HB9-tTA* plasmid was 9135 nucleotides long and identical to the *HB9* promoter sequence listed on the Addgene website (Plasmid #16275). When compared with the reference genome in the NCBI database, however, we noticed two differences: the *HB9* promoter in the *HB9-tTA* plasmid is shorter, missing three nucleotides (GGG) between nucleotides 685 and 686 and 50 nucleotides (TTAGGTTCTCTTTCTTCTTTGGGACTCTCATCTGGAAAAGGCCCATGGGC) between nucleotides 6,928 and 6,929 (Fig. 1*A*).

10.1523/ENEURO.0441-24.2025.f1-1 Figure 1-1 Nucleotide sequence of the *Tol2-HB9-tTA* plasmid. Download Figure 1-1, DOCX file.

Based on the sequence in the *HB9-tTA* plasmid, Biocytogen synthesized the *HB9-tTA* fragment and added the Tol2 transposable element and HS4 insulators at the 5′ end of the *HB9* promoter and the Tol2 element at the 3′ end of the bovine growth hormone (*bGH*) polyA signal sequence (Fig. 1*B*). In this *Tol2-HB9-tTA* transgenic vector, the *HB9* promoter sequence is 9,134 nucleotides and is missing a thymine (T) at position 8,596 compared with that in the *HB9-eGFP* plasmid, and the AscI restriction enzyme site (GGCGCGCC) located at the 3′ end of the *HB9* promoter sequence is duplicated (Fig. 1*A*). The nucleotide sequence of the *HB9* promoter and surrounding elements used to generate transgenic mice is listed in Extended Data Figure 1-1.

### GFP is expressed in dorsal horn neurons in the spinal cord of *HB9-tTA: teto-GFP* mice

Biocytogen injected the *Tol2-HB9-tTA* transgenic vector and the transposase mRNA into fertilized C57BL/6J mouse eggs, generating five founder mice. We crossed *HB9-tTA* progenies of these mice with *tetO-GFP* mice (JAX #005104) and analyzed GFP expression in the cervical region of spinal cords from 2-month-old male and female mice. *HB9-tTA: tetO-GFP* mice derived from two founders showed GFP signals in the spinal cord, which were further characterized in detail below (Line 1 and Line 4).

**Figure 2.**
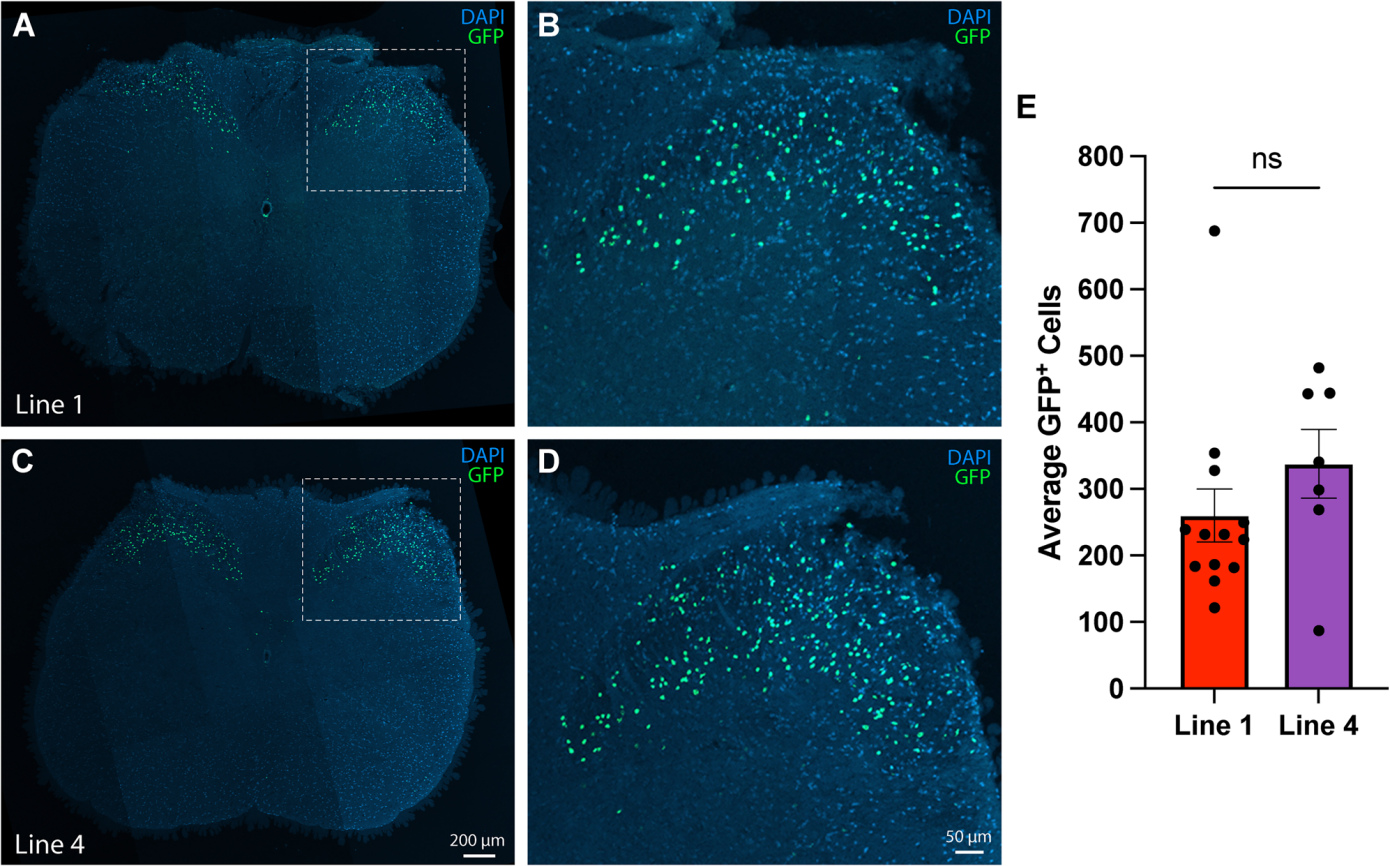
Expression patterns of GFP driven by *HB9-tTA* in the mouse spinal cord. ***A***,***C***, Coronal section of the cervical region of the spinal cord stained for DAPI. The tissues were from an *HB9-tTA;tetO-GFP* double transgenic mouse of Line 1 (***A***) and Line 4 (***C***), respectively. GFP signal was seen in a small subset of dorsal horn cells. ***B***,***D***, The area covered by the white square is enlarged fivefold to showcase endogenous GFP signal. ***E***, Quantification of the average count of GFP-positive cells within each line. Each data point represents the average of five cervical spinal cord sections from an individual mouse. The resultant data were imported to the GraphPad Prism software for subsequent analysis via an unpaired *t* test. Scale bar, 50 or 200 μm. Ns, not significant. The raw data used to quantify the number of GFP-positive cells in Figure 2*E* are shown in Extended Data Figure 2-1.

**Figure 3.**
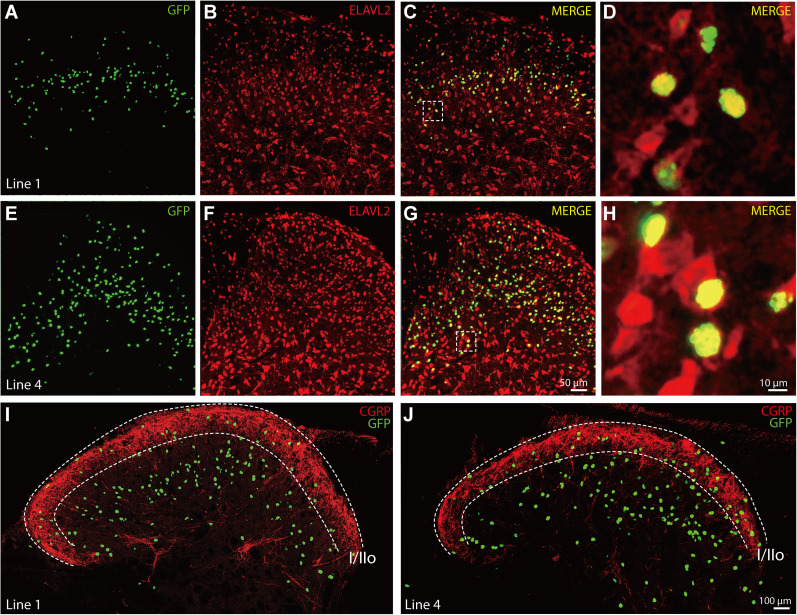
GFP-positive cells express neuron-specific marker Elavl2. Cervical spinal cord sections from *HB9-tTA;tetO-GFP* double transgenic mice were analyzed. ***A***,***E***, GFP signal. ***B***,***F***, Elavl2 immunostaining. ***C***,***G***, Merged image showcasing colocalization of Elavl2 and GFP signal. ***D***,***H***, The area covered by a white square in enlarged 10 folds to showcase colocalization of Elavl2 and GFP fluorescent signal. ***I***,***J***, Coronal section of the cervical region of the spinal cord stained for CGRP and DAPI. The tissues were from an *HB9-tTA;tetO-GFP* double transgenic mouse of Line 1 (***I***) and Line 4 (***J***), respectively. Scale bar, 10, 50, or 100 μm.

To our great surprise, in both *HB9-tTA: tetO-GFP* mouse lines (Fig. 2*A–D*), GFP was only expressed in a small subset of dorsal horn cells, but not in ventral motor neurons as we had expected. The average number of GFP-positive cells per mouse seems to be lower in Line 1 than that in Line 4 (Fig. 2*E*), based on quantification of 13 mice from Line 1 (seven males and six females) and 7 mice from Line 4 (two males and five females). The raw data for these cell counts are presented in Extended Data Figure 2-1. Immunostaining experiments revealed that all GFP-positive cells within the spinal cord were neurons, since they were all also positive for Elavl2 (Fig. 3), a neuron-specific RNA–binding protein in the mammalian brain (Ince-Dunn et al., 2012). To map their precise laminar locations, we stained spinal cord sections with an anti-CGRP antibody that labels laminar Layers I and II-outer and found that only a few GFP-positive neurons are present in CGRP-positive layers, while most GFP-positive neurons appear to be located in other layers such as II-inner and III (Fig. 3*I*,*J*), based on similar images in the literature (Xu et al., 2005; Liu et al., 2021).

10.1523/ENEURO.0441-24.2025.f2-1 Figure 2-1 The raw data used to quantify the number of GFP-positive cells in Figure 2E. Download Figure 2-1, TIF file.

### *HB9-tTA* drives GFP expression in a small number of brain neurons

**Figure 4.**
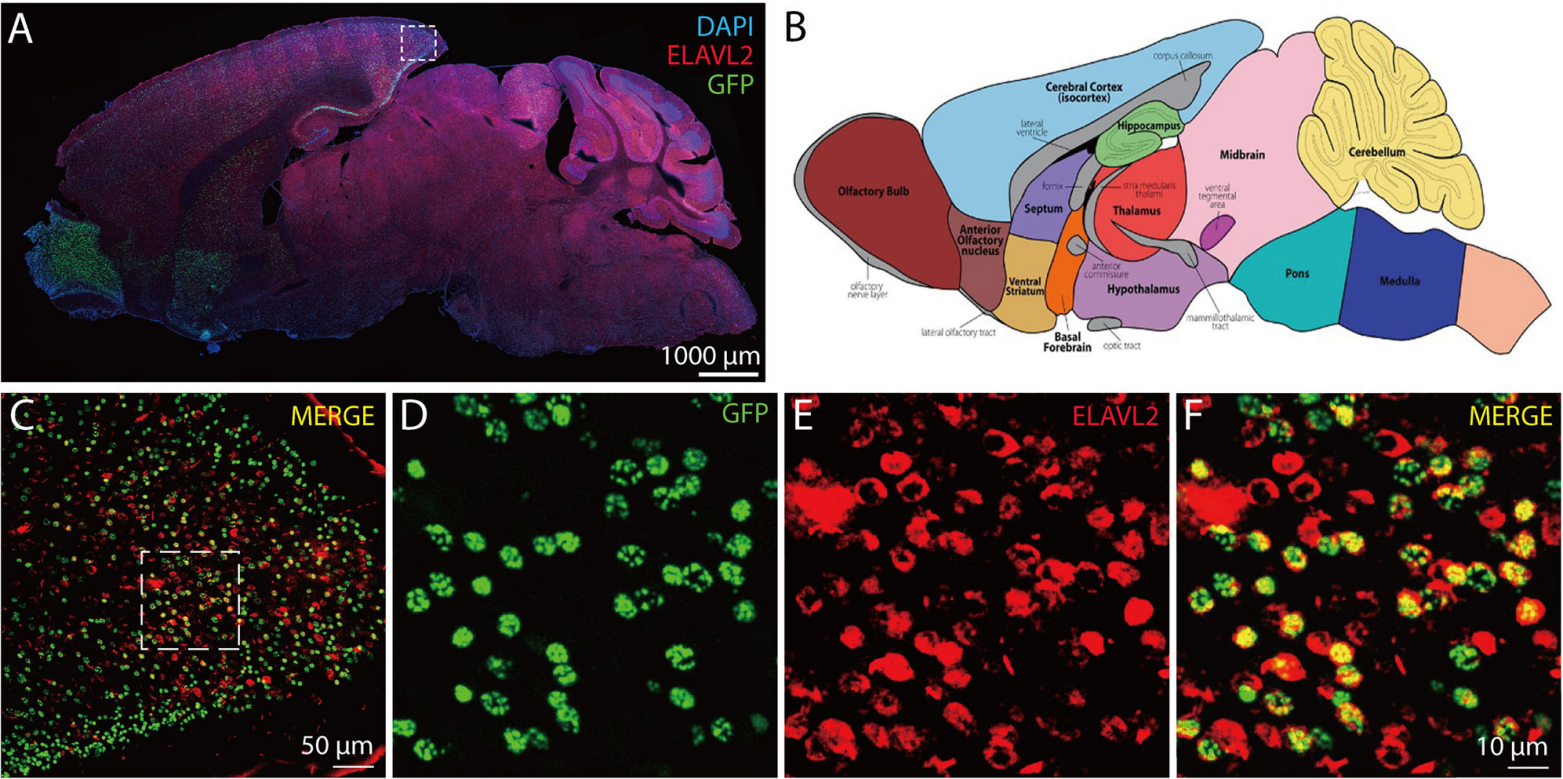
GFP expression patterns in the brain of *HB9-tTA;tetO-GFP* double transgenic mice. ***A***, A sagittal brain section of an *HB9-tTA;tetO-GFP* double transgenic mouse stained for Elavl2 and DAPI with endogenous GFP signal. ***B***, Annotated and color-coded mouse brain atlas reference image, courtesy of Rockefeller University's Gene Expression Nervous System Atlas (GENSAT) Project. ***C***, Merged image showing colocalization of Elavl2 and GFP within the posterior cortex. ***D***, Endogenous GFP signal within the posterior cortex with fivefold magnification. ***E***, Elavl2 immunostaining of the posterior cortex with fivefold magnification. ***F***, Merged image showing colocalization with fivefold magnification. Scale bar, 10, 50, or 1,000 μm. Quantification of *tTA* mRNA levels is shown in Extended Data Figure 4-1.

We then examined GFP expression patterns in the brain of a Line 4 *HB9-tTA: tetO-GFP* mouse at 2 months of age. GFP signal was prominently expressed in the olfactory bulb, ventral striatum, and the hippocampus, as well as in the anterior olfactory nucleus, cortex, septum, and posterior midbrain at substantially lower levels (Fig. 4*A*,*B*). This expression pattern seems to persist throughout adulthood, since the *tTA* mRNA expression level in the frontal cortex remained the same in 3- and 9-month-old mice (Extended Data Fig. 4-1). Very few GFP-positive cells were detected in other parts of the brain. As in the spinal cord, GFP-positive cells in the brain were also positive for Elavl2 (Fig. 4*C*–*F*); thus they are all neurons.

10.1523/ENEURO.0441-24.2025.f4-1 Figure 4-1 Quantification of *tTA* mRNA levels. Quantification of *tTA* mRNA levels in the frontal cortex of 3 and 9-month-old *HB9-tTA* mice using qRT-PCR. Compared to C57BL/6J control mice, *HB9-tTA* mice exhibit detectable *tTA* expression at both ages. There is no significant difference in *tTA* mRNA levels between the two time points, indicating that *tTA* expression is maintained in the frontal cortex through adulthood. Data are presented as mean ± SEM and analyzed by two-way ANOVA followed by Sidak’s multiple comparison tests. ***: *p* < 0.001, ns: not significant. Download Figure 4-1, TIF file.

### Identification of *HB9-tTA* insertion sites in the mouse genome

**Figure 5.**
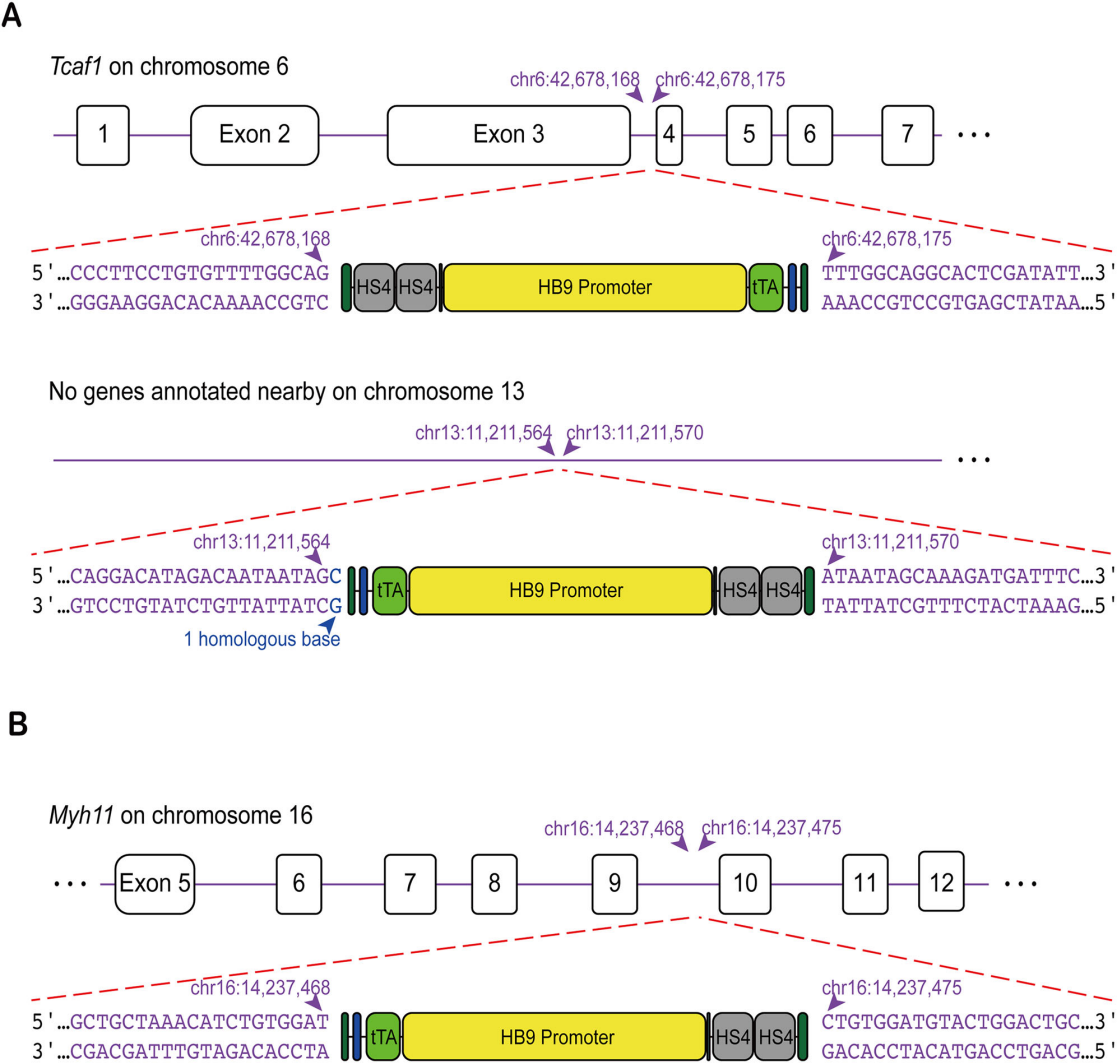
Insertion sites of the HB9 promotor in the mouse genome. ***A***, Line 1 contains two copies of the *HB9-tTA* transgene, inserted into the third intron of the *Tcaf1* gene and in a region lacking annotated genes on Chromosome 13. ***B***, Line 4 is inserted on Chromosome 16 within the ninth intron of the *Myh11* gene. Body weight and motor performance in *HB9-tTA* and C57BL/6J mice are shown in Extended Data Figure 5-1. Quantification of *Myh11* mRNA levels in *HB9-tTA* mice is shown in Extended Data Figure 5-2.

To determine genomic locations of the *HB9-tTA* insertions, primary fibroblast cells were collected and cultured, then sent to Cergentis for whole-genome sequencing. We found that Line 1 and Line 4 had the cassette inserted into different chromosomes within the mouse genome. Line 1 harbored two independent insertions: one residing on Chromosome 6 within the third intron of the gene *Tcaf1*, which encodes a protein that binds the cold-sensing channel TRPM8 and promotes its trafficking to the cell surface (Gkika et al., 2015), and the other on Chromosome 13 in a region lacking annotated genes (Fig. 5*A*). Line 4 had a single insertion on Chromosome 16 within the ninth intron of the myosin heavy chain 11 gene *Myh11* (Fig. 5*B*), and these mice maintain normal body weight (Extended Data Fig. 4-1*A*) and locomotor activity on an accelerating rotarod test (Extended Data Fig. 4-1*B*). Moreover, quantitative RT-PCR analysis showed that the *Myh11* mRNA expression level was not significantly affected in *HB9-tTA* mice (Extended Data Fig. 5-2). Taken together, it is unlikely that the host gene function is significantly affected by the insertion.

10.1523/ENEURO.0441-24.2025.f5-1 Figure 5-1 Body weight and motor performance in *HB9-tTA* and C57BL/6J mice. ***A***, Body weight (g) of eight *HB9-tTA* and eight C57BL/6J mice (equal number of males and females for each genotype at 7-month of age) was measured. Data are presented as mean ± SEM and analyzed by two-way ANOVA followed by Sidak’s multiple comparison tests. ns: not significant. ***B***, The same mice from ***A*** at the 5.5-month of age told were tested for motor coordination using an accelerating rotarod. The latency to fall (sec) was measured and each dot represents a single mouse’s average latency to fall across three total trials, with an endpoint maximum of five minutes. Data are presented as mean ± SEM and analyzed via an unpaired *t*-test. ns: not significant. Download Figure 5-1, TIF file.

10.1523/ENEURO.0441-24.2025.f5-2 Figure 5-2 Quantification of *Myh11* mRNA levels in *HB9-tTA* mice. The frontal cortex of 9-month-old *HB9-tTA* and C57BL/6J control mice was used for quantitative RT-PCR analysis. Data are presented as mean ± SEM and analyzed by two-tailed Student’s *t*-test. ns: not significant. Download Figure 5-2, TIF file.

## Discussion

The original aim of this study was to generate a mouse model in which the tTA is specifically expressed in spinal cord motor neurons under the control of the *HB9* promoter, allowing for inducible gene expression of ALS-related toxic proteins. However, the resulting *HB9-tTA* mice showed unexpected transgene expression in a small subset of dorsal horn neurons rather than motor neurons. While this outcome has deviated from our original goal, it presents a serendipitous discovery that opens new avenues for research into spinal cord dorsal horn neurons—a neuronal population that may play a critical role in sensory processing, including pain and proprioception (Todd, 2010).

One possible explanation for this unexpected expression pattern may lie in a few changes in the *HB9* promoter in the transgenic construct (Fig. 1) that may affect the binding of transcriptional factors (Spitz and Furlong, 2012). Alternatively, the insertion sites of the *HB9-tTA* transgene, which were located on different chromosomes in different founder lines, could have influenced the cell-type specificity of the *HB9* promoter and thus contributed to the restricted expression in dorsal horn neurons. This raises interesting questions about the regulatory mechanisms governing *HB9* promoter activity, which warrants further investigation.

Although this transgenic model does not fulfill its intended purpose of driving gene expression in motor neurons, it provides a unique opportunity to study dorsal horn neurons. The dorsal horn of the spinal cord plays a critical role in processing sensory information, including pain, temperature, and proprioception (Todd, 2010). Neurons in this region are integral to the transmission and modulation of sensory signals from peripheral nerves to the brain, making them a key focus in pain research and sensory disorders (Peirs and Seal, 2016). Despite their importance, few mouse models are available that allow for gene manipulation specifically in a small subset of dorsal horn neurons. One limitation of the study is the fact that the identity and connectivity of these GFP-positive neurons remains to be characterized by scientists who are interested in studying them further. Another limitation of our study is that the *HB9-tTA* transgenic vector was randomly inserted into the mouse genome. Better specificity and consistency of the expression pattern may be achieved by a targeted insertion to a noncoding genomic region. Nonetheless, the *HB9-tTA* mouse model, which offers the flexibility to temporally control gene expression, represents a valuable tool for researchers studying the development, function, and connectivity of this neuronal subpopulation.

## Synthesis

Reviewing Editor: Eran Perlson, Tel Aviv University Decisions are customarily a result of the Reviewing Editor and the peer reviewers coming together and discussing their recommendations until a consensus is reached. When revisions are invited, a fact-based synthesis statement explaining their decision and outlining what is needed to prepare a revision will be listed below. The following reviewer(s) agreed to reveal their identity: NONE.

The authors present an intriguing study detailing the unexpected creation of a transgenic mouse line targeting dorsal horn neurons in the spinal cord. While the findings are potentially valuable for dissecting somatosensory processing, the manuscript requires additional experimentation and analysis to enhance its impact and utility.

Below are specific points for the authors to address:

1. Expression Pattern in Adults. The authors demonstrated the expression pattern during development using Hb9-tTA crossed with a TetO-GFP reporter. However, it is unclear if the expression pattern persists or is significantly restricted in adults. To address this, the authors should inject a TetO-driven AAV into the adult spinal cord and characterize the expression profile at this stage. This is critical to understand the utility of this line in adult studies.

2. Spinal Neuron Characterization. A detailed histological analysis of the labeled neurons is essential. Specifically: Determine whether the neurons are glutamatergic or GABAergic by counterstaining with markers like VGluT1/2 and GAD67. Map their precise laminar locations using markers such as CGRP, IB4, or PKCγ.Provide proper quantification of these analyses, including representative images and counts.

3. Potential Functional Impact of Insertions. The authors identified two insertion sites for the Hb9-tTA transgene: the TCAF1 gene (Line 1) and the MYH11 gene (Line 4). It is crucial to assess whether these insertions influence the expression of these genes or their associated proteins. This could be done through qPCR or Western blot analysis.

4. Behavioral and Physiological Characterization. The authors should verify whether the insertion sites or the expression of this transgenic line affects the overall health of the mice. Suggested assessments include behavioral tests (e.g., motor ability, intelligence levels, and nociceptive thresholds) and physical measurements like body weight. These experiments will ensure the model's suitability for broader applications.

5. Figure 2E Analysis. The authors should label the asterisk and specify the p-value in Figure 2E. The raw data for this figure should be provided for transparency and reproducibility.

6. Data Analysis Tools. The authors mentioned using FIJI (ImageJ) for analysis but did not specify using GraphPad Prism or a similar statistical tool for Figure 2E. Clarifying this is necessary.

7. Insertion Approach. While the authors used a promoter-driven transgene insertion, an alternative approach could involve targeting a noncoding genomic region to potentially improve the specificity and consistency of the expression pattern. The authors may wish to discuss this limitation in their manuscript.

8. While the findings are novel in their unexpected labeling of dorsal horn neurons, the HB9 promoter has been widely used for motor neuron studies since its first publication over two decades ago (PubMed: 12176325). The authors should contextualize their work by acknowledging prior studies and highlighting the unique aspects of their findings.
