# Supplementary material for: New Mouse Lines That Drive Tetracycline-Controlled Gene Expression in a Small Subset of Spinal Cord Dorsal Horn Neurons
Source: eNeuro. 2025 Apr 18;12(4):ENEURO.0441-24.2025. doi: 10.1523/ENEURO.0441-24.2025 (PMC12014207; doi:10.1523/ENEURO.0441-24.2025)
Supplement: Figure 1-1 — Nucleotide sequence of the Tol2-HB9-tTA plasmid. Download Figure 1-1, DOCX file. [file eneuro-12-ENEURO.0441-24.2025-s005.docx]

Tol2-HB9-tTA plasmid sequence (TG-YK2-001))

Tol2 left or right arm

5' HS4 insulator sequence

T3 promoter

HB9 promoter

tTA sequence

(T), missing T

bGH poly(A) signal

restriction enzyme sites

CAGAGGTGTAAAGTACTTGAGTAATTTTACTTGATTACTGTACTTAAGTATTATTTTTGGGGATTTTTACTTTACTTGAGTACAATTAAAAATCAATACTTTTACTTTTACTTAATTACATTTTTTTAGAAAAAAAAGTACTTTTTACTCCTTACAATTTTATTTACAGTCAAAAAGTACTTATTTTTTGGAGATCACTTCATTCTATTTTCCCTTGCTATTACCAAACCAATTGAATTGCGCTGATGCCCAGTTTAATTTAAATAGATCTGGCCATCTAGACATGGAATCGATGTCAGGTACCGAGCTCACGGGGACAGCCCCCCCCCAAAGCCCCCAGGGATGTAATTACGTCCCTCCCCCGCTAGGGGGCAGCAGCGAGCCGCCCGGGGCTCCGCTCCGGTCCGGCGCTCCCCCCGCATCCCCGAGCCGGCAGCGTGCGGGGACAGCCCGGGCACGGGGAAGGTGGCACGGGATCGCTTTCCTCTGAACGCTTCTCGCTGCTCTTTGAGCCTGCAGACACCTGGGGGGATACGGGGAAAAAGCTTTAGGCTGAAAGAGAGATTTAGAATGACAGAATCATAGAACGGCCTGGGTTGCAAAGGAGCACAGTGCTCATCCAGATCCAACCCCCTGCTATGTGCAGGGTCATCAACCAGCAGCCCAGGCTGCCCAGAGCCACATCCAGCCTGGCCTTGAATGCCTGCAGGGATGGGGCATCCACAGCCTCCTTGGGCAACCTGTTCAGTGCGTCACCACCCTCTGGGGGAAAAACTGCCTCCTCATATCCAACCCAAACCTCCCCTGTCTCAGTGTAAAGCCATTCCCCCTTGTCCTATCAAGGGGGAGTTTGCTGTGACATTGTTGGTCTGGGGTGACACATGTTTGCCAATTCAGTGCATCACGGAGAGGCAGATCTTGGGGATAAGGAAGTGCAGGACAGCATGGACGTGGGACATGCAGGTGTTGAGGGCTCTGGGACACTCTCCAAGTCACAGCGTTCAGAACAGCCTTAAGGATAAGAAGATAGGATAGAAGGACAAAGAGCAAGTTAAAACCCAGCATGGAGAGGAGCACAAAAAGGCCACAGACACTGCTGGTCCCTGTGTCTGAGCCTGCATGTTTGATGGTGTCTGGATGCAAGCAGAAGGGGTGGAAGAGCTTGCCTGGAGAGATACAGCTGGGTCAGTAGGACTGGGACAGGCAGCTGGAGAATTGCCATGTAGATGTTCATACAATCGTCAAATCATGAAGGCTGGAAAAGCCCTCCAAGATCCCCAAGACCAACCCCAACCCACCCACCGTGCCCACTGGCCATGTCCCTCAGTGCCACATCCCCACAGTTCTTCATCACCTCCAGGGACGGTGACCCCCCCACCTCCGTGGGCAGCTGTGCCACTGCAGCACCGCTCTTTGGAGAAGGTAAATCTTGCTAAATCCAGCCCGACCCTCCCCTGGCACAACGTAAGGCCATTATCTCTCATCCAACTCCAGGACGGAGTCAGTGAGAATATTCTCGACGGTACCGAGCTCACGGGGACAGCCCCCCCCCAAAGCCCCCAGGGATGTAATTACGTCCCTCCCCCGCTAGGGGGCAGCAGCGAGCCGCCCGGGGCTCCGCTCCGGTCCGGCGCTCCCCCCGCATCCCCGAGCCGGCAGCGTGCGGGGACAGCCCGGGCACGGGGAAGGTGGCACGGGATCGCTTTCCTCTGAACGCTTCTCGCTGCTCTTTGAGCCTGCAGACACCTGGGGGGATACGGGGAAAAAGCTTTAGGCTGAAAGAGAGATTTAGAATGACAGAATCATAGAACGGCCTGGGTTGCAAAGGAGCACAGTGCTCATCCAGATCCAACCCCCTGCTATGTGCAGGGTCATCAACCAGCAGCCCAGGCTGCCCAGAGCCACATCCAGCCTGGCCTTGAATGCCTGCAGGGATGGGGCATCCACAGCCTCCTTGGGCAACCTGTTCAGTGCGTCACCACCCTCTGGGGGAAAAACTGCCTCCTCATATCCAACCCAAACCTCCCCTGTCTCAGTGTAAAGCCATTCCCCCTTGTCCTATCAAGGGGGAGTTTGCTGTGACATTGTTGGTCTGGGGTGACACATGTTTGCCAATTCAGTGCATCACGGAGAGGCAGATCTTGGGGATAAGGAAGTGCAGGACAGCATGGACGTGGGACATGCAGGTGTTGAGGGCTCTGGGACACTCTCCAAGTCACAGCGTTCAGAACAGCCTTAAGGATAAGAAGATAGGATAGAAGGACAAAGAGCAAGTTAAAACCCAGCATGGAGAGGAGCACAAAAAGGCCACAGACACTGCTGGTCCCTGTGTCTGAGCCTGCATGTTTGATGGTGTCTGGATGCAAGCAGAAGGGGTGGAAGAGCTTGCCTGGAGAGATACAGCTGGGTCAGTAGGACTGGGACAGGCAGCTGGAGAATTGCCATGTAGATGTTCATACAATCGTCAAATCATGAAGGCTGGAAAAGCCCTCCAAGATCCCCAAGACCAACCCCAACCCACCCACCGTGCCCACTGGCCATGTCCCTCAGTGCCACATCCCCACAGTTCTTCATCACCTCCAGGGACGGTGACCCCCCCACCTCCGTGGGCAGCTGTGCCACTGCAGCACCGCTCTTTGGAGAAGGTAAATCTTGCTAAATCCAGCCCGACCCTCCCCTGGCACAACGTAAGGCCATTATCTCTCATCCAACTCCAGGACGGAGTCAGTGAGAATATTCTCGAGGATCCGCGGCCGCGAGCTCAATTAACCCTCACTAAAGGGAGTCGACTCGATCCTGTCTCCCTGGCCAGGAGTCTGGGGTCCGCAGATATATTACAACAAAATTACTAATTGGAAAATGAAATTGTCAATAAAGCACACCCCACGCGTGTACTATATCTTTAAATATTATGATGTCTTATTGGTCCCATTTCCTTACAATAATTTATTGCAATAAAACAGACACTGCAGGTCCCCTTAAATTGTCTTTTCATAATGAATAAATTTAAGCAGGCTAATTAATATATAAACTAGCTCAATTTGTCAAGTTGATTTGTATTTTAGTTAATTGTGAAAGTAATTACCACATGGTCAAATTAACAGCTTTCTGGAAATGACCAAGCCTGAGGTTTTATTTCCTTCCTGGGTGAAGAAAATTCATTTTTCCAAGCTCTTGATGTGATGAATAAAAGTCATAAATCTGGGTGATTGGTGCAGGCAGAGTCTAAATGGCTTCATATTTCATTTTAGGTTTAATAGAAATATTCATGCTCTGTTTTAATGAAATTAAATTGAAGGGGGATGGGGCTCTGGAGGCGGCCCTGGCTGGGGGCTTAAAGGGACAGTGCACTTTTCCTTTCTTCATGCAAGGGCCAGGGCTAGAGGTCCCTGGACTCAGTCAAGAAGTGGAGAGAGCTGAGGGAGGCCTGGGAGGCCCTTATTCTGTACCCAGTGTAGCACACCTGCAGCCCTGATTGGGGGGGGGGGGCGGGGGCAATTCACACCCTGTATTCTCACCAGGCTAAAGTTTCTTCTATCTGGTTCCTTCTCTCCAAAAAATCAGTCAGAAGGGACCTCCCTTGGCTGAGAGTAGAAGTGAGGTGGTCAGCGTGCTGCAGACAAGCCTGTCCTCCATCTGCCTAAGGGGTCTGATATCCTACACCAAGCCCCCCCTACCCCTTCTGGAGACACATAGGAGATAAGCATTTTCAAAACCACCATGGCACTCTCCTGTTCTCCCCTGCACCCCTTCCTCCCAGACTCCATCCTGCCTGAGGATGATTTGGTCCAACTTGAATAATAGAGTACTCTCTTTCCACATCTTTCCACATCGGAAAGAGTAGTCCGGGATCCAGTCCACCTGCAGCCCTGATTAAAGGAAGGAAGGAAGACTAGAGGCGCAGTTCTACCTATTATACTTATTTTAAGGTTCCCAGGTCAGAGATGCCTCATGGACATTTCTCCCTAAGGCAGGTCCTGACCAGCCCTCCCCCTGAATGGTGCTGATCTGTGACTTGTTGCTTCTATTGAACAAATACAGCTTTGATTTGTTTCCAGCCAGAGTGGTTAGCTGATGAATTGACAAAAACTAATCAGCTTTATTGGGAAACAGGTTTAAGGGCACGGACGTGTCAATAACGCTCAGCCTGACCCCCTCTTCCATTAGCTAGGCAGGCTGATTAGAGTAAGAAGACCCTCTCGCCGATTGCACCGAAATTGCTTTCATAAACTCACAATATTAGCAGTCGATGTGAGCAGCTAATTTAGAGAAGACATCCAGGCCCGTCACATCGATCATCGGGTTTATGTATCATTATCTCATTTACAGAATGTCGCAGTGTAATACATGTTTTTGGCAATAGGCTTGGCCGCTCGCCGGAGCCGCGCGCTGTTCCTTTCGGCCACACTGCAGGGGACTTCCTCAACAATCTACGATGTCTCTTCGCGTGAACAAGGAGGCACTTTGCACACATATGCACACTTGCGCCCAGGCTCTCAGTGCGCGCTCAGTGCACACATGCACGTGGACACATGCTCCGACATACTACCTGAGCTGCCTTTCCTTCTCATGCGGGGATGCTGGCATTGCTCTCTCCTGCGAGCCCCTGGTCTGAAGCCCAATGCCGGACGCTCACCAACCTTGGAGTCAACCTCGGAGAGCTCAAGGTTCGGTTCTGTCACCAGCATACAGAGATGGTGGGTGGCGGCGCAATCAGCCACCTTCTCTCAAGGCTTTGGTCGCCCAGTTGGCGGGTGTCCAGAAATCCACAGGCCTGCGAAGGTCCTTCTCCGCCCTCCCAGGCTCCAGCTTGACCTGCCCTGCCCTGGGCGGGCCTTTCTCGCAACACTTCCAGGCTCAGCCAGATGCGCCTGGTCTTAGGGAGTGGGCCACCTGTTCCTTTGGGTAATTATTTTAATGTAAGGGGTCTACTTCAGGCCTCCTGCAGTGACAGACGGCAGCGGAGCTGCAAAGCGCTCTCAGCCTTTCAGTGGAATTTCCCAGATGGGCCAAGGGGAGGCCAGGGCGGGTCACCAGGGGCCCTTTAACGAGTTCATTAGCCAAATTATCTTGCCTGCAGCAAGGACCCCAGAGACTGTAGGGAGAGTAGGGGAGAAGGTGGGTCTTTCTCCCTCCCTGCTAGTCTGCAAGAACAGTTGTGGGAAGGAAAGAGAAAGGACATCCTCCCCAAAGGGGCCTAGGAACACTAAAGGCAAAGATGGCATCTCTGATGTGACTTATGCGCCAGACCAGCGAGGTGGTGGAGTGGTGGGAAGGAATCATAATGATTAGGAAACACGGAGATTCTAACAACCAGGGCACTGACCCATATTGTGGAACAAACCTAGTGCAGAAATCAAAGTCAGAGCCCCTGGAACTTTATTCTGTGCTTCTTAAGCAAAGAACCTGGACATCTTGGAGCCTTGGTCTGTCCGTCCATCTGTAGAGTAGAAGGTAGGTGTGTTTCCTTCCACAATTTGTGAGATCACTGCCCTAAGAAAAGCAACTCTGGGAAGAGCTTCTGGCATGACCCTGTTGGGTCCTTCGGGCCCCCTTACTCCTGTGGTAGGAGGCTTTGGCAGGTTGAGGTGAGCCTGATCCTAGATGCCTGTGCTGGAAAATGAACCCAACTCTCACCTGTCGTCGCGCCCCCCACCCCCACCCCACAGCTGTGAGGCGCTGCTCTGGAGGTAGCTCAGCATCCCCGCCCCTCCTCCTTGTCCTCACTCTGCCTCCTGTAGTGTTAGCTTCTGCTACCTCAGCAAGTAGTGAGTGAATAGACCAATTAGCCAGGACTTCCTGCCATTATCTGCTGTAGACTTGGGCATTAGTACGCCCTGATTCACCATAATTTCAAGTCAGAACACACTAAAAAATTCACTCACCGAGGCATTAAAATGTTCCCAGACAACGTCTGTGTTTGTGTAACTGGGTTTCTTCCCTTCGTGTTTGATGCACACCAGCATCATATAGGTCAGTGGTGGAGTCCAGGCTAGGCAGACAATTTTTGTCGCTTTTTGTTTGTTTTTATATTTTTAAAACATGAATTGCATTGTTTCCCGGTGGTTGTAAACTTTGTTTCTGGGGCTTCAGTATTGCCAGTGTAATCTTCTTCTGCTTGATCAGGGGCAAACAAGTGACTTAGAAAGTTGTGGGAGTGCCCTAAGAACAGTAGCCAGAGCCTTTACAGAGCCAGGCTTTGTGTCCTAAACGTACATCGCCCCAGCTATGGGAAGCTGGGTTATCACCTTCAAAGCAGACCCTTTCTGTGGATGACTTACCAAGCCCTGCAGGTGGGTAGTGGAGGTGGGGAGAGGGCTGCACAGCCTGCTTGGAGCTTGCTTCTGTTCTTTGTCCTGCCCATGATATGAAGCTCCTATACAATGAATTTGCAGTAAGATCTTGGAGCAAAAGTACAGGTTTCCTGAGAAGTCTTTCGTTTCTTCTGAGGGTGCTGTTTTACTTACACACACACACACACACACACACACACACACACACACACACACACCCTAGCTGATTAAAAGAACTGAGGTGACCTCTCTTAGCCTTGTTTCTCTTTATGATTCATACCTATGGCCTCTAGGATTGTAAGAGAAATTGAAAATGTCTTTGGCTTCCTGGGTCAGAAAGCATCCAGCGCAGGGCTGAGCTCAGTCTCTTCTCTATGTTCTAAGTACACAGCAAAACAAACAAAAACCCGAAACCTTGAGTAAAATTTTCAAATATAGAATATATTTTTGTGCTGGAGTTTTTCAAATTTATATCTTACTGTCTTTCTCCCTGTACCCCTACAACCCAGGTTTGGGCTGGACTTATTTATTTATTTACAAGGAGGAATGGGGCCAGTGGAGAAGGGCAGCCAGTGAAGATGGCCTGAGGGTGGACCAGGCAGTAGGGAGGGCAGAGTAAGCTTTCTAAAAAGGGAGGAAGGAAATCCAAGTGTGCTGGGAGAGGTAGAGAAGGGGGGGGGGCTGGCAAAGAAAAGGACTGGCTTGTAGTGTCCTTGGCAAGAGCAGTTCACCTCAGGTTGGAACACTGGGTCTTCAGGTCACCTGTCTTCTGAGCCACCTTTGCAAACCACCCCTTTAAAGCTGAACTGGGGATGGAGGTCTGAGAAAAGGGTGGTAGATCAAAGGATGGTAATAAAGGAGCTGATGGTACTTTTGGGCTCTGAAGGGGCTGTAATGAGTGTGTGTGTGTGGGGGGGGACTCATCAGACATCCCTTCTCCTGATCTCACCTCCCTGTGACCCCAGGCTCCCCATTGGATTCTGTGTGTCCTCAGGCTGGGGTGGGTGGACGGGTTATGTCCCCAGAGACATTCCAAGCACTGATTTCAGTCTGACTTGAAGGAAGTTGGTCTATGACCTTCCAGGTCCAGCATTCACACTTCCTGCTTGCTTTCTGTCCTCCTGAGTACTTATTCAGCCTCTGAAAAATACCACCATCACCACCCCTGTGGGCTTGTGTCCTGGACCTCACTGGCCTCACTACATCCTCCAGGCTCATTTTCCTGAAGGGAAGCCTTAAAATGTTTTTTTTTTTAAAGATTTATTTATTTATTATATGTAAGTACATTGTAGCTGTCTTCAGACACACCAGAAGAGGGAGTCAGATCTCGTTACAGATGGTTGTGAGCCACCATGTGGTTGCTGGGATTTGAACTCTGGACCTTCGGAAGAGCAGTAGGGTGCTCTAACCCACTGAGCCATCTCACCAGCCCCGCCTCAAAATGTTGATGTGAGTTAACTCTCATGGAGTTCAAAAGGGCTGAGAAGTCAGTCTTCATGAACCTTTCTCCATGAGGCTGCTGTGGGCAGGGGGTTGCTTGTTTTAAGTTTTGGAAACAGCCAGACAAATGGCAGGTGTGTGGAGGGAGATGCAGGGAATGCTGCCTTCCATCCAGTCTCCTAGCCATACCTCCAGGCTCTTGGCCTTTTTGTTAATGGCTATTATATATTGTACCTGCTGCATTCCACAGAAAGGGATGTGTTTGTAGGTGATCAGTTATAGAATCAATGCTGACCTGACCTGTGAATATGGGTGCATGTAGGTTTTGTTCTGAGTTCTTGTATAATGTCAGGGGTAAAACTTGTAGATTATAACATCTTTGTAAGTTTTCTTTTTATGTTTGCATTGTGTGTATGTTTGTGTCTATTTATGGGAATGCACGTACCCAAAGAGGCCATAAGAGGGAATCCGATTTCCTGTAGCTGGAGTTGGAGGTGGTTGTGAGTGTGAGTGGTGGGAACCCAACTTGGGTCCTCCTCAAGAGCTGCACTCACTCTTAAGCACTGAACTGTGGAACCACTATACTCCCCACCCCAACATCTTATTTCCTTGTGGTTATTTGTATAAATCAACACACCTTTCTCAGTAATGAGAGGAGGCTCAATCACAAGTTATATTCAGTAACTTAATGCAAGACCCAAAAGAGGGATCCTGGCTAGACGCAGAGAAAAAAATGACTGGGAGCCAGATGATTAAATGTACACCGGGCCTTAAATTCCAGCCTCTTTTGGGTAGGAGAGGTCTTCCTCTCTGCCCTCCAAGAGCCTCATTTGGCCCCTGGGGCTTCTGGAGAAGGGAGCTACCTTGAGGCACAACAATGGGAATGCTGTGCTCACTTCGGCAGCACCTATACTAAAACTGAACCGATACAGAGAAGATTAGCATGGCCCCTGCGCAAGGATGACATGCAAATTTGTGAAGCATTCCTTATTTTTTTTTGCCAGGACCAGGAATGGGAACAGGTGGGTTGGGGAGCAGAGGGAGGGGAGAGGGGATAGGGGATTTGGGGAGGGGAAACTAGGAAAGGGGATAACATTTGAAATGTAAATAAAGAAAATATCTAATAAAAAAAAAAAAAAGAATAGGCATGCTTCTGGGACCAGACCTTTGATCTCTCAAGGGTACCGTAGTAAACCCCCAGCTCAGTCCCAGGCTGCCCCAGAGCCAGCTGGGCTGGGTCAATAGACGCTGGGAACAGGAAGAGACGCAGCATGCTCACTCCACTCGTAAAACACACAAAGATCCCAGTTCCAGACGACTCCCAGTCTCTCTCCCAACTCCCACCTCTCAGGATACAAGTTCCCGCTCAAACCGAAGCAGAATATATGATCATATTCAAGCCCTTTGACTTCAGAGTCTTTTTGGCCCAACCCCCACTTTGTCAAACAAAGAAATATATCCATCAAGTCTGATATGCAAATTTCCCCTTCGCTGAAGGGGCTCTTGATACAACCTCACTCAGCCCACAGGTGGTGGGGACTGGTGTGGGTTAGCCTGGTGGATGGTCAAACACCAGACGAAGTGGGAAGGGTTTGAAAGGGATTTAGAAGCTTTTGGATCACATCCCCTACACAACAAACAGGAAATGAATTCCTCTTCTCGGATGACTTTACTAAGGAAACCTGTGCCTAAACTCAGTAGACCTAATACTGTATCATCACCAAAAACAACCCTGGTTGTGTAGTTTAGAGATGATTCGGGTCCATGAGGTCCACCCCAGGAAAATCTCATCTGCAGCCACCCTTTTTTTCCTCCTTCTTCTCCTTTGTTTGTTTTGTTGTTGTTTTTTTCAAGACAGGGTATCTCTGTGTAGCATTTTTGGTTGCTGTCCTGGAACTGGCTCTGTAGACTAGGCTGGCCTAGAACTCACAGACCCCACCTGCCTCTGCCTCCTGAGTGCTGGGATTAGAGACTTGCCTCACCATGGCTTAAACACAAGTTTCTGTAAATGCAATGGGGTCAAGACTGAATCCTGGGGATGGGGGTAGGGCATCCCAGCATCCTTACTTGCCTTAATTGGGCTTCCAAGATAGGAGAACTGGCAGGGAACCACTCATGGAACACAAGTGTTCCTTTAACCCTCTAAAAGGCAGAGCTGCATTTTCATCACCCCAAAGAGACAGCACCTAAAGACAAGAGCTATGTGCTTGTACTATTTTCAGACTAGCTGGAGGCCTTTGCAAAGCTGTGCACAACTTACTCCAAGATGTTCCCCGCTGGGTTCCAGATTTTCCAGCCAGGACCACCCTGTCATAATAAAAACTCCACTCAGCCGAGCATGGTGGCGCACACCTTTAATCCCAGCACTTGGGAGGCAGAGGCAGGAGGATTTCTGAGTTCGAGGCCAGCCTGGTCTACAGAGTGAGTTCCAAGACAGCCAGGGCTAGACAGAGAAACTCTGTCTCCAAAATCAAAAACAAAAACAAAACAAAAACAACAAAAACAAAAAACCTACTCACTCATCCCAACAGAATCACTGCGTTAAATCTCTCCTAAACAAAACTTGTTGACAGGGAAAATATAGTCCTTACTTATATTTATCGTTACCCCCCACCCCCAATCCCTCTTGAAACAGCTTTGCCTGCTACTTTTAAATTCACCCAACAAGGATTGTCCTAGCCTCCTGGCCTTCCCTGGGCCATTTCCGATCTTTTATTAACCTCTCACCTGGTTGGGAAGCCGCGATCCAGAGACCTGGAACTCCCTCCCACTTTCCCCTCGTGTTTGGGGAAGATACCCGAGAATGGAGCTAGCGAGAAAGCTTGATTTCTCAAACTACTTATTGATTCGTTACATTAAAAAAAAAAAGCCCCCGGAAGCGTGTTTACAGTAAATATTTGATATGTGCGCAGGCTGAGGTCTGGATTCACACTTTAATACGGTCTCTGGAAGCCCTTTCTTGATTTTCCTGAACTCGGCCGGCAGGAGGAGGCGAGAGCCTGATAGTGGCTCCCTTAGCCTGCGAACTGGCTCAGTCCGAGGGAGCAGTCCTCGGCCAATTCCCGCAAAGCCTTTTTCCTACACCCCCACAAGGCAAGGCTCTAATAGAACTTGGTTCTCACCTAGGTCCGGCTCAGTGGCACCTGTGCAAACGACCTTCAAGTCAATGTGCATCTCTGAGTTGGAAAACTGCTCTCTCTCCACTCTGACTGAAGATTTGTAGGCTTCAACCTCTAATACCGTATTTATACACACCGAAACCCTACAAACAAACATTTGCGGCGCATGATACTTTTGGGGGAGGGGCAGATAAGAAGGTTCGACCTGCTGAGGGTTAATGACAGGGACGCAAACCATCTTGAAGCAATCAACACTCAGGAACGCGAGAGATTTTGGGAAACCGAACAGTCTTCAGTGTACTAAGCTCAGGGTCCCGTTTTGGGTACCTAGTGGACTTCAGGGGTGAGGACCCACCTAGTTCCTCAGTGCAGAACATTCCACAACTCCCTCAAGGGAGCGCGAGTAGCTGGAGGAAGATAAGGCGTCCGCCTCTGGAGGAACAAGGTGTCAAAGGGACTCGGGGGGCACTCACTGGGACCCAGGGTTACTGGCGCTCACCACTCTCCCAGCAGCAAGGAAGCAGTAAGCTTGTGT(T)CAGCCTCCAAACTCTCCAACCTGCCTCCTCCGGCCACCTACGCTGTTCACCACCGGCTGCTTAGGACCTGGGGGCGCAGGGGCGGGGCGTCAGGCTAATTGGACCGTGACAGGTGAGCCCCTCGGCCAATCGCCGGGCAGACAGCCCTCCTCGGGCCCCCGCTGAGCGCGCCAATCCCAGACCGCCCTGAGCTTCAAGTGTGCGGAGGGGCGCGGGTCCCCACCAAGGCACCAGTGGCCTCGATGCGGTCGCTTGAGCTCTTGAGAGCAGAGAGCCGGTCTCCCCAACACAAACTCGCAGGAGCGCTCCGGTGGAAGTTCATACCAGTGAGTGGAGCGCGGTCCCCACGAGGACCGCAAAGAGGAGGCGCCTGGACGATGGTCGCTCTCAGCTGAGTTTCCGGCTGCGACTTTATTGGCAAAAAATCGCAGTAACAATACCGGCCCCAAGGCTGGCCACCGCCACGCTCAGTCTCCGTAAGCCCGCACACGACTGCATCGCCCACCCTTGGACCCTGATTGTCAGCTCCAATCGAGCCGGCGCGCCGGCGCGCCATGTCTAGATTAGATAAAAGTAAAGTGATTAACAGCGCATTAGAGCTGCTTAATGAGGTCGGAATCGAAGGTTTAACAACCCGTAAACTCGCCCAGAAGCTAGGTGTAGAGCAGCCTACATTGTATTGGCATGTAAAAAATAAGCGGGCTTTGCTCGACGCCTTAGCCATTGAGATGTTAGATAGGCACCATACTCACTTTTGCCCTTTAGAAGGGGAAAGCTGGCAAGATTTTTTACGTAATAACGCTAAAAGTTTTAGATGTGCTTTACTAAGTCATCGCGATGGAGCAAAAGTACATTTAGGTACACGGCCTACAGAAAAACAGTATGAAACTCTCGAAAATCAATTAGCCTTTTTATGCCAACAAGGTTTTTCACTAGAGAATGCATTATATGCACTCAGCGCTGTGGGGCATTTTACTTTAGGTTGCGTATTGGAAGATCAAGAGCATCAAGTCGCTAAAGAAGAAAGGGAAACACCTACTACTGATAGTATGCCGCCATTATTACGACAAGCTATCGAATTATTTGATCACCAAGGTGCAGAGCCAGCCTTCTTATTCGGCCTTGAATTGATCATATGCGGATTAGAAAAACAACTTAAATGTGAAAGTGGGTCCGCGTACAGCCGCGCGCGTACGAAAAACAATTACGGGTCTACCATCGAGGGCCTGCTCGATCTCCCGGACGACGACGCCCCCGAAGAGGCGGGGCTGGCGGCTCCGCGCCTGTCCTTTCTCCCCGCGGGACACACGCGCAGACTGTCGACGGCCCCCCCGACCGATGTCAGCCTGGGGGACGAGCTCCACTTAGACGGCGAGGACGTGGCGATGGCGCATGCCGACGCGCTAGACGATTTCGATCTGGACATGTTGGGGGACGGGGATTCCCCGGGTCCGGGATTTACCCCCCACGACTCCGCCCCCTACGGCGCTCTGGATATGGCCGACTTCGAGTTTGAGCAGATGTTTACCGATGCCCTTGGAATTGACGAGTACGGTGGGTAGACCGGTCCTGCAGGGTTTAAACGGCCGGCCATTGACTAGTGGATCCCATGGGGGCCCGCTAGCCCTAGAGCTCGCTGATCAGCCTCGACTGTGCCTTCTAGTTGCCAGCCATCTGTTGTTTGCCCCTCCCCCGTGCCTTCCTTGACCCTGGAAGGTGCCACTCCCACTGTCCTTTCCTAATAAAATGAGGAAATTGCATCGCATTGTCTGAGTAGGTGTCATTCTATTCTGGGGGGTGGGGTGGGGCAGGACAGCAAGGGGGAGGATTGGGAAGACAATAGCAGGCATGCTGGGGATGCGGTGGGCTCTATGGCTTCTGAGGCGGAAAGAACCAGCTGGGGCTCGAGTCTAGTTTAATTAAGGCGCGCCGAATTCCATGGATATCAAGCTTAAACAAGAATCTCTAGTTTTCTTTCTTGCTTTTACTTTTACTTCCTTAATACTCAAGTACAATTTTAATGGAGTACTTTTTTACTTTTACTCAAGTAAGATTCTAGCCAGATACTTTTACTTTTAATTGAGTAAAATTTTCCCTAAGTACTTGTACTTTCACTTGAGTAAAATTTTTGAGTACTTTTTACACCTCTG
