# Supplementary figures and images for: New Mouse Lines That Drive Tetracycline-Controlled Gene Expression in a Small Subset of Spinal Cord Dorsal Horn Neurons
Source: eNeuro. 2025 Apr 18;12(4):ENEURO.0441-24.2025. doi: 10.1523/ENEURO.0441-24.2025 (PMC12014207; doi:10.1523/ENEURO.0441-24.2025)

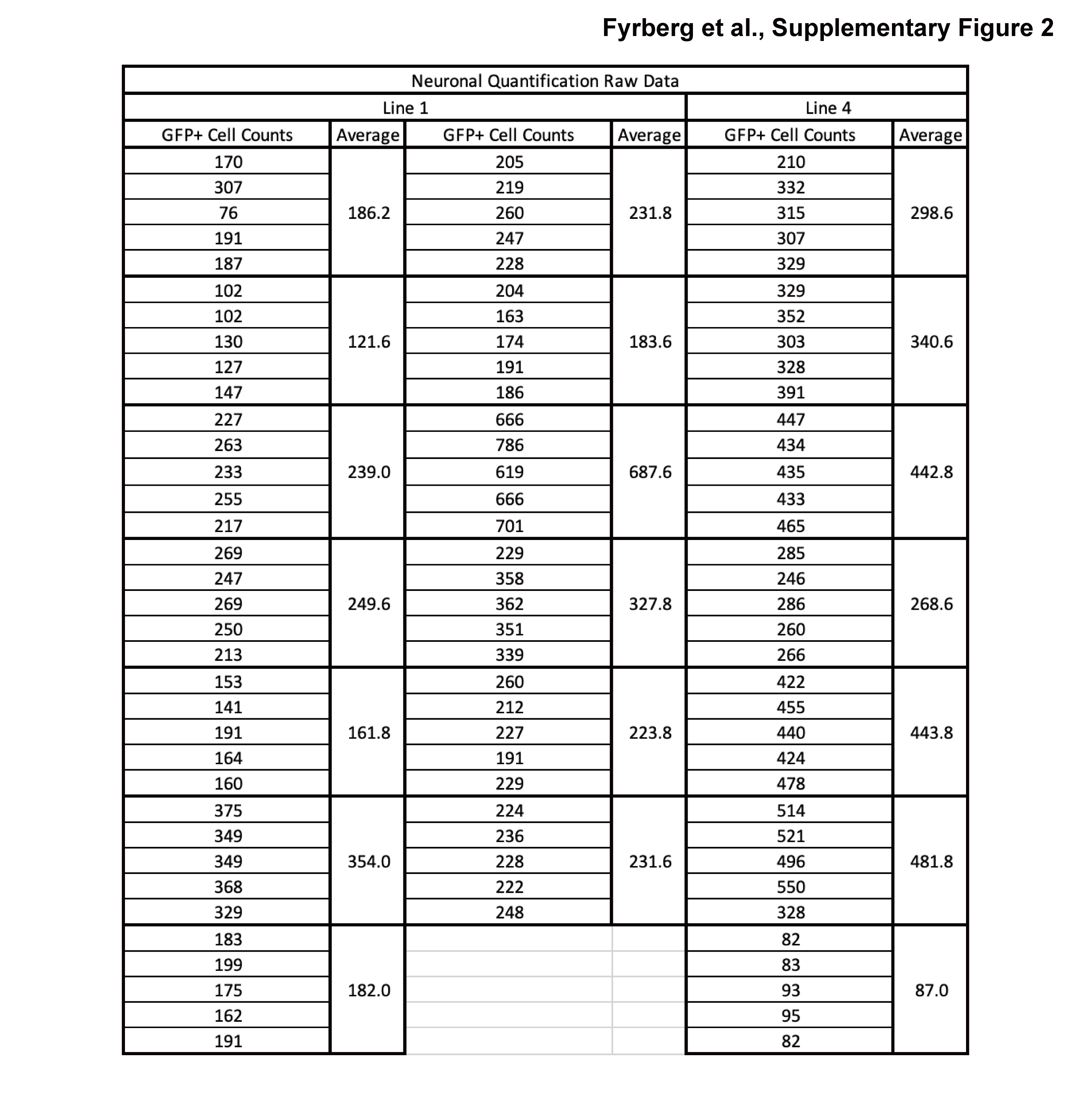

Supplement: Figure 2-1 — The raw data used to quantify the number of GFP-positive cells in Figure 2E. Download Figure 2-1, TIF file. [file eneuro-12-ENEURO.0441-24.2025-s001.tif]

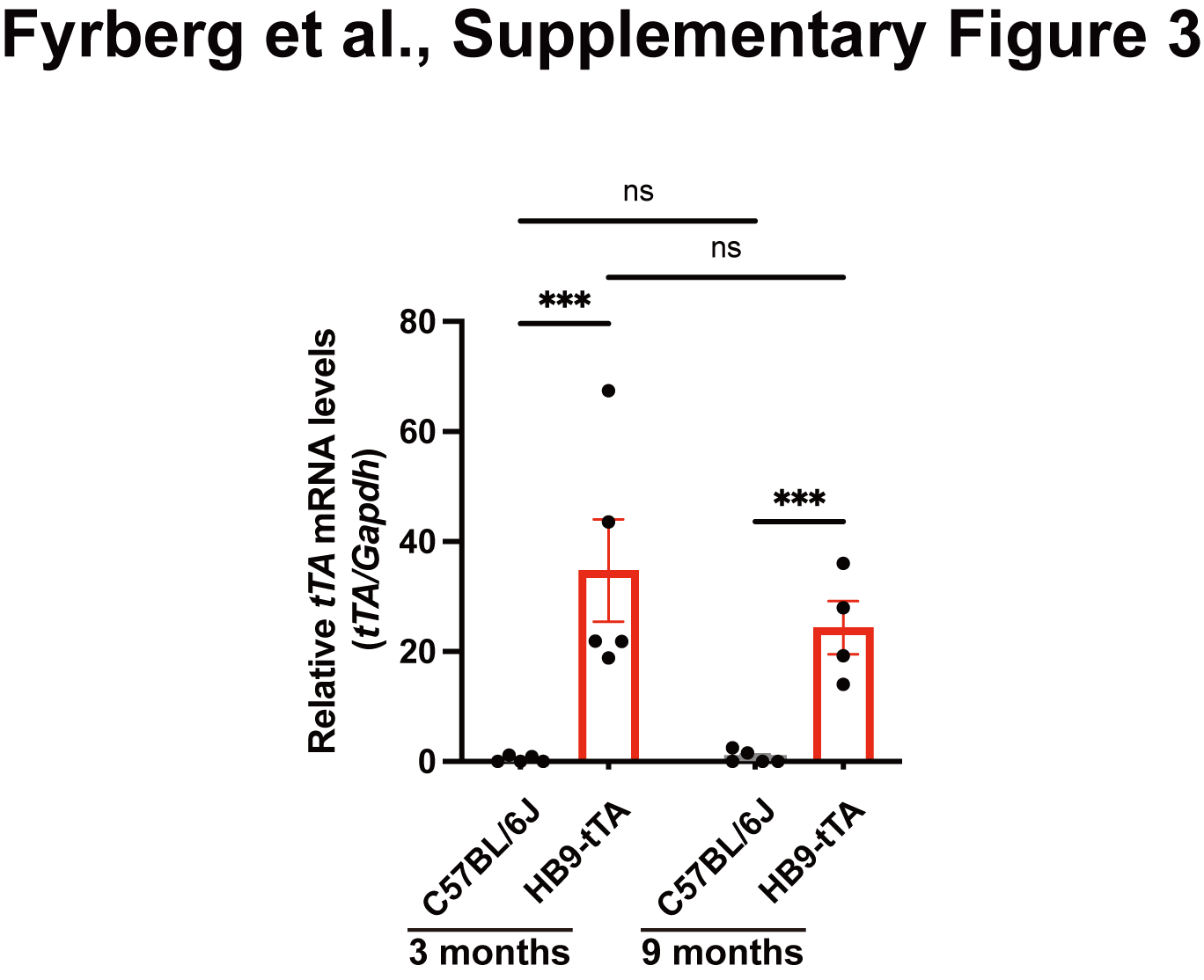

Supplement: Figure 4-1 — Quantification of tTA mRNA levels. Quantification of tTA mRNA levels in the frontal cortex of 3 and 9-month-old HB9-tTA mice using qRT-PCR. Compared to C57BL/6J control mice, HB9-tTA mice exhibit detectable tTA expression at both ages. There is no significant difference in tTA mRNA levels between the two time points, indicating that tTA expression is maintained in the frontal cortex through adulthood. Data are presented as mean ± SEM and analyzed by two-way ANOVA followed by Sidak’s multiple comparison tests. ***: p < 0.001, ns: not significant. Download Figure 4-1, TIF file. [file eneuro-12-ENEURO.0441-24.2025-s002.tif]

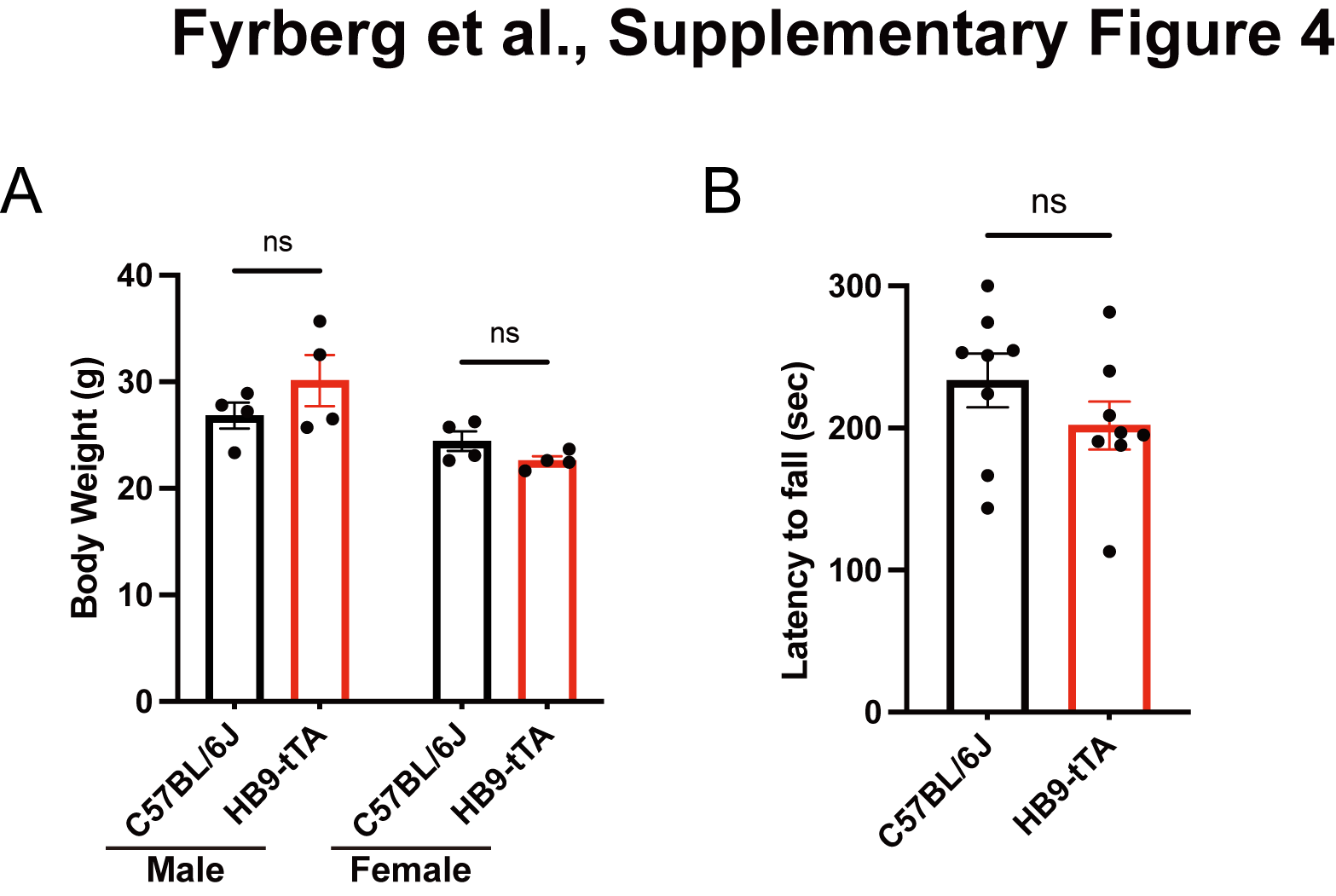

Supplement: Figure 5-1 — Body weight and motor performance in HB9-tTA and C57BL/6J mice. A, Body weight (g) of eight HB9-tTA and eight C57BL/6J mice (equal number of males and females for each genotype at 7-month of age) was measured. Data are presented as mean ± SEM and analyzed by two-way ANOVA followed by Sidak’s multiple comparison tests. ns: not significant. B, The same mice from A at the 5.5-month of age told were tested for motor coordination using an accelerating rotarod. The latency to fall (sec) was measured and each dot represents a single mouse’s average latency to fall across three total trials, with an endpoint maximum of five minutes. Data are presented as mean ± SEM and analyzed via an unpaired t-test. ns: not significant. Download Figure 5-1, TIF file. [file eneuro-12-ENEURO.0441-24.2025-s003.tif]

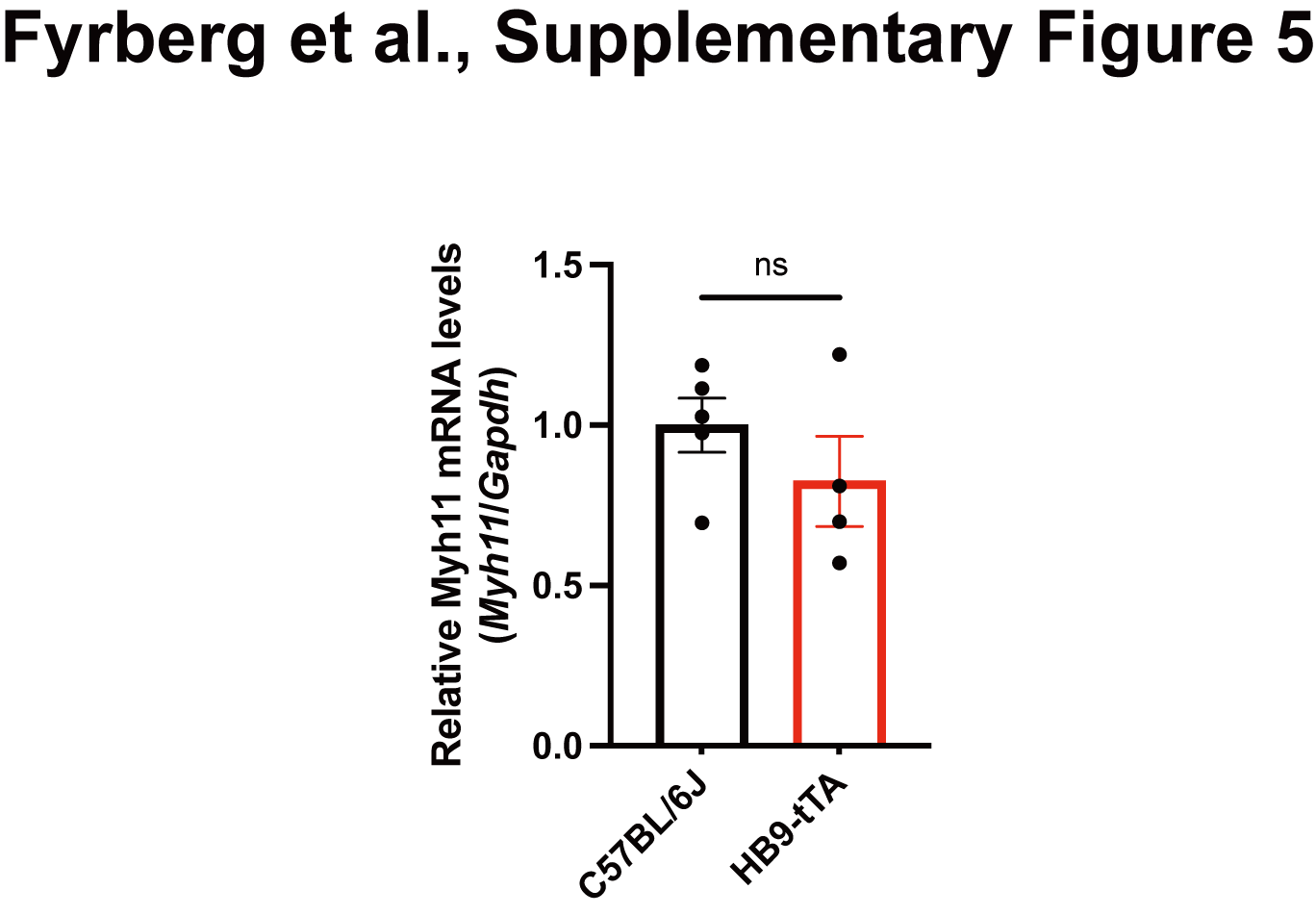

Supplement: Figure 5-2 — Quantification of Myh11 mRNA levels in HB9-tTA mice. The frontal cortex of 9-month-old HB9-tTA and C57BL/6J control mice was used for quantitative RT-PCR analysis. Data are presented as mean ± SEM and analyzed by two-tailed Student’s t-test. ns: not significant. Download Figure 5-2, TIF file. [file eneuro-12-ENEURO.0441-24.2025-s004.tif]
